# Artificial intelligence for patent ductus arteriosus—a systematic review

**DOI:** 10.3389/fped.2026.1648943

**Published:** 2026-01-30

**Authors:** Sarah Elizabeth Long, Theodor Uden, Corinna Peter, Steffen Oeltze-Jafra, Philipp Beerbaum

**Affiliations:** 1 Department of Pediatric Cardiology and Intensive Care, Hannover Medical School, Hannover, Germany; 2 Department of Pediatric Neonatology, Pulmonology and Allergology, Hannover Medical School, Hannover, Germany; 3 Peter L. Reichertz Institute for Medical Informatics, Hannover Medical School, Hannover, Germany; 4 CAIMed: Lower Saxony Center for AI & Causal Methods in Medicine, Hannover, Lower Saxony, Germany

**Keywords:** artificial intelligence (AI), deep learning, ductus arteriosus, machine learning, patent ductus arteriosus, PDA, PROBAST

## Abstract

**Introduction:**

Optimal management of patent ductus arteriosus (PDA) remains controversial. Complexity in severity appraisal, high-dimensional data, and the need for longitudinal, individualized assessment make PDA a compelling candidate for Artificial Intelligence (AI)-driven approaches. This systematic review evaluates AI research in the context of PDA, identifying strengths, limitations, and future directions.

**Methods:**

Following PRISMA 2020, databases were searched for peer-reviewed articles from January 1, 2010, to May 31, 2025. Eleven studies met inclusion criteria. Data on design, population, sources, AI methods, performance, validation, limitations, and explainability were extracted. Risk of bias was assessed using the Prediction model Risk of Bias Assessment Tool and Joanna Briggs Institute Critical Appraisal Checklist; reporting quality using the Minimum Information about Clinical AI Modeling checklist. Heterogeneity precluded meta-analysis; therefore findings were synthesized narratively.

**Results:**

Eleven studies addressed diagnosis/screening (*n* = 5), treatment-response prediction (*n* = 2), risk-factor identification (*n* = 2), treatment-complication prediction (*n* = 1), and subphenotype analysis (*n* = 1). Ten were retrospective; nine single-center, one multi-center, and one used a national registry. Sample sizes were mostly <500 (range: 66–8,369). Definitions of PDA subgroups—symptomatic and hemodynamically significant PDA—varied significantly. Populations included preterm, neonatal and pediatric cohorts, often excluding other congenital heart disease, pulmonary hypertension, or early mortality. Input data ranged from multimodal parameters to high-dimensional unimodal sources. Ten studies used supervised learning; nine traditional machine learning; five deep learning. No study performed adequate external validation. Diagnostic models achieved AUCs of 0.74–0.93, however risk of bias was high, particularly in analysis, suggesting overfitting. Models addressing other aspects showed modest performance. None of the included studies exhibited low risk of bias. Most studies addressed explainability to some degree; only one addressed clinical utility; none evaluated fairness. Reproducibility was hindered by manual preprocessing and limited sharing of data, models, or code.

**Conclusions:**

Artificial intelligence shows feasibility for supporting PDA risk stratification, diagnosis, severity assessment, and prediction of treatment-related outcomes. However, current applications remain in early, pilot-stage development and are not yet suitable for clinical implementation. Future work should prioritize clinically meaningful tasks, scientifically rigorous and bias-aware methodologies, larger and more representative cohorts, and systematic external validation. Fairness, explainability, and reproducibility must be addressed to support translation. Continued methodological refinement and clinical grounding will be key to unlocking the potential of these technologies for this highly vulnerable patient population in the future.

## 1 Introduction

### 1.1 The clinical problem

Patent ductus arteriosus (PDA) describes the persistence of a fetal blood vessel connecting the aorta and pulmonary artery beyond 72 h after birth, resulting in abnormal circulation (1).

Despite decades of research efforts, PDA assessment and management remain challenging (2). Depending on the context, direction and volume of shunting, a PDA may be protective—as in duct-dependent congenital heart disease or pulmonary hypertension—a benign bystander, or hemodynamically significant. In contexts of early postnatal discharge or limited healthcare resources, a PDA may go undetected, only discovered at neonatal readmission due to complications or incidentally (3).

In the case of hemodynamic significant PDA, excessive left-to-right shunting can lead to pulmonary over circulation and systemic steal, which is associated with serious comorbidities in preterm neonates, including chronic lung disease, intraventricular hemorrhage, necrotizing enterocolitis, heart failure, pulmonary hypertension, and increased mortality (4, 5).

The hemodynamic impact of a PDA exists on a continuum; while some cases can be readily deemed as insignificant or significant, many fall within intermediate states that defy binary classification. Further complicating clinical assessment, the hemodynamic status of a PDA is inherently dynamic and spontaneous closure can occur. In the first days and weeks of life, shunt direction typically shifts from right-to-left or bidirectional—due to elevated pulmonary resistance—to left-to-right as pulmonary pressure falls (2). A PDA that initially appears insignificant may progress to hemodynamic significance, while one that seems likely to require intervention may begin to close spontaneously. About one-third of infants under 1,000 g experience spontaneous closure within the first 2–6 days (6), and around 47% of very preterm infants within the first year (7).

As such, longitudinal evaluation is essential to monitor the PDA's evolution over time. Currently, serial echocardiography remains the gold standard for evaluating PDA, providing cross-sectional snapshots that assist clinicians in determining whether the ductus is likely to close spontaneously or progress toward hemodynamic significance (8).

To mitigate subjectivity and facilitate longitudinal appraisal clinicians have traditionally relied on the comparison of unidimensional parameters over time, such as measurements of PDA diameter, LA:Ao ratio and the presence of retrograde diastolic flow in abdominal arteries. However, many of these parameters are subject to considerable intra- and inter-observer variability and demonstrate limited correlation with PDA-associated comorbidities (9).

As a result, despite decades of research, diagnostic criteria for hemodynamic significant PDA remain non-standardized. Five main categories of echocardiographic parameters can be found in the literature—duct assessment, pulmonary overcirculation, systemic hypoperfusion, end-organ perfusion, and myocardial performance—with considerable variation in which parameters are selected, how they are weighted, and cut off thresholds across institutions (4, 8, 10).

To support PDA assessment via echocardiography, clinicians also consider diverse multimodal data (4), including but not limited to ventilator dependency, comorbidities, cardiac stress biomarkers like BNP and N-terminal pro-BNP (11, 12), and imaging tools for additional insights into systemic perfusion such as renal and cerebral Doppler ultrasound and near-infrared spectroscopy (NIRS) (4).

More recent efforts to capture PDA severity in a standardized manner include a variety of echocardiography-based PDA severity scores, summarized in Table 1. Multiple scores demonstrate high predictive performance on their internal validation sets, but face challenges in clinical adoption, likely due to the inclusion of parameters that are not routinely measured, a lack of external validation and, on the receiving end, heterogeneous institution-specific practices (10). Furthermore, the frequent exclusion of neonates with additional congenital heart disease from development and validation cohorts, together with reliance on data from high-income countries, limits the generalizability of these scores to neonates with congenital heart disease comorbidities and to populations in low- and middle-income countries.

**Table 1:** Echocardiography-based PDA severity scores ordered from left to right based on year of publication.

Score	McNamara-Sehgal Score (52)	El-Khuffash et al. PDA Severity Score (PDAsc) (35)	Shaare Zedek Score (53)	Umapathi et al. PDA Severity Score (PDAss) (36)	Iowa PDA Score (54)	PLASE Score (55)
Predictive Outcome	CLD	CLD/death	CLD/death	CLD/death	death <36 weeks or severe BPD	Surgical ligation of PDA
Timepoint of Parameter Acquisition	Day of Ibuprofen therapy	2nd day of life	2nd day of life	Within 7 days of life	Between 12 and 24 h of life	3rd day of life
Study Population for Score Development	52 preterm neonates with GA < 32 weeks who received Ibuprofen for PDA closure; 27 developed CLD	118 preterm neonates with GA < 29 weeks and open ductus arteriosus on day 2; 65 developed CLD, 15 died before discharge	Derived from common practice at Shaare Zedek Medical Center (SZMC), validated via El-Khuffash cohort	98 preterm neonates with GA < 32 weeks; 34 developed CLD, 2 died before discharge	Derived from protocol at University of Iowa, applied in cohort of 73 preterm neonates < 24 weeks GA as part of assessment of early hemodynamic screening impact	692 preterm neonates with GA < 30 weeks; 77 required surgical ligation
Country of Origin	Australia	Ireland, Canada, and Australia	Score from Israel, data from Ireland, Canada, Australia	USA	USA	Japan
Parameters	PDA diameter; Max PDA velocity; PDA:LPA diameter; PAedv; LPAedv; LA:Ao ratio; LV:Ao ratio; LVO:SVC; Mitral E/A ratio; IVRT	GA; PDA diameter; Max PDA velocity; LVO; LV a’ wave	PDA diameter; LA:Ao ratio; DFR; PDA Doppler shunt pattern	GA; PPI; LVO; SMA VTI; PV Vd; DFR in dAo	PDA diameter: weight; LA:Ao ratio; PV D wave; LVO:RVO; Mitral E/A ratio; IVRT; DFR in dAo, celiac, or middle cerebral artery	GA; PDA diameter; LPAedv; LA:Ao
AUC	0.91 (95% CI, 0.83–1.00)	0.92 (95% CI: 0.86–0.97)	Not reported*	0.97 (95% CI: 0.93–0.99)	Not reported**	0.827 (0.744–0.911)
Sensitivity	88.90%	92%	Not reported	94%	Not reported	Not reported
Specifity	88%	87%	Not reported	93%	Not reported	Not reported
PPV	88.90%	92%	Not reported	94%	Not reported	Not reported
NPV	88%	82%	Not reported	93%	Not reported	Not reported

PDA, Patent ductus arteriosus; CLD, Chronic lung disease; BPD, Bronchopulmonary dysplasia; LPA, Left pulmonary artery; PA, Pulmonary artery; edv, End diastolic velocity; LA, Left atrium, Ao, Aortic root; LV, Left ventricle; GA, Gestational age; PPI, pulmonary perfusion index; LVO, Left ventricular output; SMA VTI, Superior Mesenteric Artery Velocity Time Integral; PV, Pulmonary vein; Vd, Peak diastolic flow velocity; DFR, Reversal of flow in diastole in descending aorta; IVRT, Isovolumetric relaxation time; RVO, Right ventricular output; SVC, Superior vena cava; Mitral E/A ratio, Mitral inflow velocities; a’ wave, tissue Doppler atrial contraction wave; PV D wave, pulmonary vein diastolic wave.

* Correlation with El-Khuffash PDAsc in El-Khuffash cohort 0.62, *p* < 0.001; Correlation with CLD/death in El-Khuffash cohort: *p* = 0.02.

** Utilized as part of an early hemodynamic screening protocol which demonstrated a two-fold reduction in the composite primary outcome of death prior to 36 weeks or severe BPD in the <24 weeks GA cohort.

In attempting to standardize PDA severity assessment—whether through individual parameters or scoring systems—clinicians must reduce highly complex, multidimensional data into simplified metrics. This process, though necessary for human assessment, risks overlooking the complexity of PDA, is examiner dependent, and may direct clinical attention toward parameters that are not reliably predictive of relevant outcomes.

Establishing evidence-based treatment guidelines requires a body of research that demonstrates methodological consistency for reliable comparison and validity. Without such a foundation, it is unsurprising that consensus is lacking regarding which preterm infants benefit from treatment, when intervention should occur, and which therapeutic strategy is most effective (13).

Current treatment options include cyclooxygenase (COX) inhibitors (14), transcatheter occlusion devices, and surgical ligation (2). Historically, PDA management strategies have included prophylactic (15, 16), early targeted (17), and conservative approaches (18). In recent decades, there has been a marked shift toward expectant management (19) and transcatheter occlusion devices for definitive closure (2). This change has been driven by several factors: concerns about adverse effects of COX inhibitors, incomplete response to pharmacological closure attempts, risks associated with surgical intervention, high rates of spontaneous closure (6), and inconclusive findings from randomized controlled trials (RCTs) examining pharmacologic PDA treatment. Jansen et al. (20), analyzing 47 RCTs, found a potential reduction in severe IVH with early indomethacin but no consistent benefits otherwise, while Hundscheid et al. (21), reviewing four RCTs, reported no significant differences in outcomes between conservative and active management. Similarly, Mitra et al. (22), based on 14 RCTs, concluded that early treatment likely does not reduce mortality or major morbidities. However, reviews were limited by moderate to low certainty of evidence.

Experts hypothesize that treatment may still be beneficial, but only in a well-defined subgroup—namely, infants with hemodynamically significant PDA—and that the inconclusive results of RCTs likely reflect underlying limitations in study design (2, 4). Many trials have relied on binary classifications—distinguishing simply between the presence or absence of PDA—thereby overlooking the nuanced spectrum of hemodynamic significance. Others, while aiming to focus on hemodynamically significant cases, employed heterogeneous and insufficiently validated criteria to define this subgroup (23). Furthermore, high rates of rescue therapy in control groups may have further diluted observable differences. In the absence of adequately stratified evidence, current data remain insufficient to support the development of evidence-based treatment guidelines.

This uncertainty is particularly consequential given the high prevalence of PDA in preterm infants—affecting roughly 30% of those born at 32 weeks’ gestation and as many as 70% at 25 weeks (24). These extremely vulnerable neonates often present with a complex, individualized constellation of comorbidities. As such, assessing treatment necessity requires careful consideration, not only of the PDA's hemodynamic significance, but its impact within each infant's broader physiological context.

Without standardized criteria or guidelines, severity appraisal and treatment decisions continue to rely on case-by-case expert judgment and institution-specific protocols (25). This contributes to inconsistencies in patient care, which is highly dependent on access to experienced sub-specialists in pediatric cardiology and neonatology—ultimately disadvantaging patients in resource-limited settings. Furthermore, the heterogeneity in clinical practice contributes to a self-perpetuating cycle: it undermines the design and comparability of randomized controlled trials which continues to impede the establishment of evidence-based treatment guidelines (26).

Viewed as a whole, PDA represents a highly complex condition within an exceptionally vulnerable patient population, requiring individualized, context-aware clinical decision-making. Despite ongoing efforts, current approaches remain limited in their ability to capture the multifactorial, dynamic nature of PDA pathophysiology. It is precisely these challenges—diagnostic ambiguity, high-dimensional data, and the need for individualized assessment—that have led researchers to consider PDA a compelling candidate for artificial intelligence (AI)–based approaches (27). The application of AI to identify relevant risk factors, support diagnosis, and guide management represents a relatively new and promising frontier in PDA care.

### 1.2 AI—a tool for improvement?

Unlike traditional methods that rely on predefined, low-dimensional feature sets, AI systems can process the full spectrum of available data, without the need for prior dimensionality reduction. In the context of echocardiography, for example, AI can analyze every pixel of every frame in a video sequence, leveraging precise spatial and temporal information, the complexity of which would be inaccessible to human interpretation (28). This computational depth enables AI to discover complex high-dimensional patterns—an attractive capability given the nonlinear and multifactorial nature of PDA and neonatal physiology. By extracting relevant features and capturing temporal dynamics across diverse data modalities, AI has the potential to enhance PDA diagnostic precision, improve early risk stratification, and support real-time, individualized clinical decision support (27). Importantly, these systems can do so without the limitations of inter-observer variability or the scarcity of highly specialized clinical expertise (29).

Despite these promising benefits, it is important to note that most clinical AI systems remain in the early stages of development. Such models are currently limited by oversimplified training data, narrowly scoped tasks, modest performance metrics, limited external validation, and insufficient readiness for real-world clinical integration (30). Unlike general AI applications, which can leverage vast publicly available datasets (e.g., natural images or text), medical AI development is constrained by limited access to large, high-quality clinical data. This scarcity—driven by ethical, legal, and logistical barriers—makes model training substantially more challenging (30). Additionally, the high capital investment required to meet computational demands has led to significant disparity between proprietary industry models and those developed in academia.

Beyond these challenges, there are also fundamental limitations in how AI systems operate. They identify mathematical patterns based on the data they are trained on. As a result, their accuracy and reliability are closely tied to the quality, diversity, and representativeness of the training data (30). Moreover, the complexity of AI models—particularly those based on deep learning architectures—often renders their decision pathways non-transparent for humans, limiting interpretability for clinicians. The field of explainable AI (XAI) seeks to address this by developing tools to clarify model behavior, but achieving effective, clinically relevant explainability remains an ongoing challenge (31).

Nonetheless, despite these current limitations, AI remains a promising avenue for advancing PDA management. To support meaningful progress, it is important to assess the current state of research, evaluate its contributions and shortcomings, and identify priorities for future work.

### 1.3 Objective

This review aims to systematically synthesize and evaluate current research on the application of AI in the context of PDA, including all aspects of risk assessment, diagnosis and management, identifying trends and directions for future research.

### 1.4 Existing knowledge

In their 2023 correspondence on McAdams et al.'s review (32) of AI in neonatal care, Sharma et al. (27) address the underexplored domain of AI for PDA, particularly hemodynamically significant PDA in preterm infants. They suggest that AI could enhance decision-making and outcome prediction, especially for pharmacologic treatments, by integrating imaging data (e.g., echocardiograms)—a notable advance over earlier clinical prediction models reliant on logistic regression and basic clinical data. They briefly reference two relevant studies [Lei et al. (33); Na et al. (34)] that will be discussed in more detail in this review. Sharma et al. (27) identify evaluating treatment necessity and predicting therapeutic response as desirable AI applications, noting that current scores for PDA severity assessment [El-Khuffash et al. (35); Umapathi et al. (36)] show promise but lack widespread use. The authors argue that AI could refine such scoring systems, enhancing early diagnosis and severity appraisal and paving the way to modeling treatment responses in the future. Concluding their correspondence, Sharma et al. (27) concur with McAdams et al. (32) that AI's predictive capabilities in neonatology are still developing. They propose that it holds substantial promise for PDA management and is likely to become as essential to patient care as physical examinations and laboratory testing. As a correspondence rather than a systematic review, Sharma et al. (27) did not aim to comprehensively cover all studies on AI in the context of PDA. Furthermore, since its publication in January 2023, further research in this area has emerged.

### 1.5 Relevant AI background information

To provide context for the subsequent exploration of AI applications in PDA, this section briefly outlines relevant concepts in artificial intelligence.

Artificial Intelligence (AI) refers to computer systems designed to perform tasks that typically require human intelligence. A core method in AI is machine learning (ML), where systems improve their performance on a given task by inferring patterns from training data rather than following explicitly programmed rules. This “learning” process begins with rough guesses and improves iteratively by adjusting internal parameters to minimize error during training.

At the heart of any machine learning system is a model—a mathematical structure that represents the relationships between inputs and outputs, optimized for a specific task. In traditional machine learning, these models are often relatively simple and rely on clearly defined features. For example, a model might use gestational age, ventilator dependency, and lab values to estimate the risk of hemodynamically significant PDA. These inputs are selected and structured by humans, and the model learns how to weigh them based on the data.

One example of a traditional machine learning model is the decision tree, which generates predictions by iteratively splitting data into subsets using decision thresholds optimized to best separate the training data for a given task. At each split, the model selects the feature and threshold that most improve the quality of the split according to a predefined mathematical criterion, forming a hierarchical sequence of if–then rules that terminate in leaf nodes representing final classifications or predictions. While simple and interpretable, individual decision trees can be prone to overfitting—meaning they may capture noise instead of underlying patterns, reducing their accuracy on new, unseen data. To address this, ensemble methods—such as Random Forest, XGBoost (Extreme Gradient Boosting), and Light Gradient Boosting Machine (L-GBM)—combine the predictions of multiple decision trees to improve accuracy and generalizability. These methods are particularly effective for structured tabular data and can model complex, nonlinear relationships.

While these models are well-suited for structured input, other types of data—such as medical images or unstructured clinical notes—require more advanced techniques. This is where deep learning (DL) becomes particularly valuable.

DL is a more advanced approach within ML that uses models with many layers—known as neural networks—to automatically learn complex features from raw data. Instead of requiring manual feature selection, DL models can process unstructured data directly. For instance, in echocardiography, early layers in a neural network might detect simple visual patterns like edges, while deeper layers recognize chambers, vessels or lesions. This layered structure enables DL models to capture patterns that traditional models might miss.

Before learning can take place, raw data—such as medical images, electronic health records, or clinical text—must be converted into numerical form in a way that reflects its structure and meaning. The exact method depends on the type of data and the task. For example, the pixels in an image are typically represented as matrices of intensity values; categorical labels like disease types are encoded as numbers; and words in clinical notes are transformed into vectors that capture semantic relationships—so that, for instance, “fever” and “temperature” are closer in vector space than “fever” and “fracture.” These numerical formats enable the model to carry out operations that highlight useful patterns.

As the data passes through each layer of the model, it undergoes transformations: values are multiplied, added, and passed through functions that emphasize relevant features while suppressing noise. The model contains many parameters, which are gradually adjusted during training to reduce errors, minimizing the difference between a predicted and ground truth value. A model type refers to the general category of ML approach and a model architecture specifies the particular design and structure within that type—for example, how many layers it has, how they are connected, and how information flows through them. Hyperparameters are model settings that are defined before training (e.g., learning rate, tree depth etc.) and influence how the model learns. These are distinct from model parameters, which are optimized during training. Methods such as Grid search systematically test combinations of hyperparameters to find the optimal configuration for a given model and a given task.

Models are often trained using supervised learning, where they are given input-output pairs—for example, echocardiography cine loops labeled with PDA yes or no—and learn to associate features in the input with the correct output. In unsupervised learning, the model is given no labels and must find patterns in the data on its own. For instance, analyzing thousands of patient records, it might group patients with similar lab results and symptoms who respond to the same treatments or develop similar complications. These groupings—called clusters—can reveal unknown disease subtypes or treatment responses without prior instruction.

Once trained, the model is used in inference, where it applies what it has learned to new, unseen data. To ensure reliability, models are evaluated using standard performance metrics—quantitative measures of how well they perform. Common metrics include accuracy (how often predictions are correct), precision (how many positive predictions are correct), and recall (how many actual cases are detected). Another widely used metric, AUC (Area Under the Receiver Operating Characteristic Curve), summarizes the model's ability to distinguish between different classes. To assess performance fairly, data is typically split into three parts: a training set to train the model, a validation set to fine-tune it during training, and a test set to evaluate final performance. This approach helps avoid overfitting—where a model memorizes training data instead of learning general patterns.

In addition to internal testing, external validation is often performed using entirely new data—from different hospitals, populations, or time periods—to check how well the model generalizes to real-world clinical settings. Both internal and external validation are central to ensuring the model's predictions are not only accurate but also robust and clinically trustworthy.

A key challenge in clinical AI is explainability—the ability to understand how a model arrives at its predictions. This is especially important in medicine, where clinicians must be able to justify decisions and maintain accountability. Traditional ML models, such as decision trees or logistic regression, are often more interpretable because their decision pathways are based on a small set of predefined features. In contrast, DL models learn complex, high-dimensional representations that are distributed across many layers, making their internal logic far less transparent and more difficult to interpret. To address this, the field of explainable AI (XAI) is developing tools to make models more understandable. XAI techniques aim to make complex predictions more interpretable by showing how input data influences the output. These include feature attribution methods, such as Shapley Additive Explanations (SHAP), which assign each input feature a value representing its contribution to a specific prediction. SHAP does this by comparing the model's output across many combinations of features to determine how each one affects the result. For image-based models, outputs can be interpreted using visual explanation methods such as Grad-CAM++ and other saliency maps, which highlight image regions most influential to a given prediction, offering intuitive, spatial insight into the model's reasoning. While these tools do not fully restore human-level interpretability, they offer valuable insights that can help clinicians assess whether a model is behaving reasonably.

Training effective models in medicine also involves addressing data-specific challenges. One common issue is class imbalance, which occurs when one category (e.g., presence vs. absence of disease) is significantly more frequent than others in the training data. This can bias the model toward the majority class and reduce performance for minority cases. Specialized techniques, such as reweighting or oversampling, are used to mitigate this.

In settings where high-quality labeled data is limited—a common challenge in medical AI—researchers use techniques designed to improve model performance and generalizability despite data scarcity. One such method is data augmentation, which artificially expands the dataset by generating plausible variations of existing data, such as rotating, flipping, or adding noise to medical images. This encourages the model to learn underlying patterns rather than memorizing specific examples, enhancing its ability to generalize. Another widely used strategy is transfer learning, where a model initially trained on a large, general dataset is adapted to a more specific task. Instead of training a new model from scratch, this approach leverages previously learned representations and fine-tunes them for the target application.

## 2 Methods

### 2.1 Literature search strategy

This systematic review followed the Preferred Reporting Items for Systematic reviews and Meta-Analyses (PRISMA) 2020 (37) guidelines to identify studies of the application AI in the context of PDA. A comprehensive search was conducted across PubMed (742 results), Cochrane Library (295 results), and IEEE Xplore (135 results), with supplementary searches using tools such as Connected Papers and ResearchRabbit. The final search was completed on June 13, 2025. The search terms combined AI-related keywords (“AI”, “Artificial Intelligence”, “ML”, “Machine Learning”, “DL” “Deep Learning”, “Neural Network”, “Algorithm”, “Automated” or “Computer-Assisted”) with PDA-specific terms (“PDA”, “Patent Ductus Arteriosus”, “Persistent Ductus Arteriosus” or “Ductus Arteriosus”). Filters were applied to include peer-reviewed journal articles published in English between January 1, 2010, and May 31, 2025.

### 2.2 Study selection and eligibility criteria

Titles and abstracts were screened to assess relevance. A total of *n* = 14 articles describing the use of AI in the context of PDA published in English between January 1, 2010, and May 31, 2025, were identified. Exclusion criteria encompassed non-peer-reviewed articles, correspondence letters, and conference abstracts lacking sufficient methodological detail, leading to the exclusion of *n* = 2 publications (27, 56).

**Figure 1:**
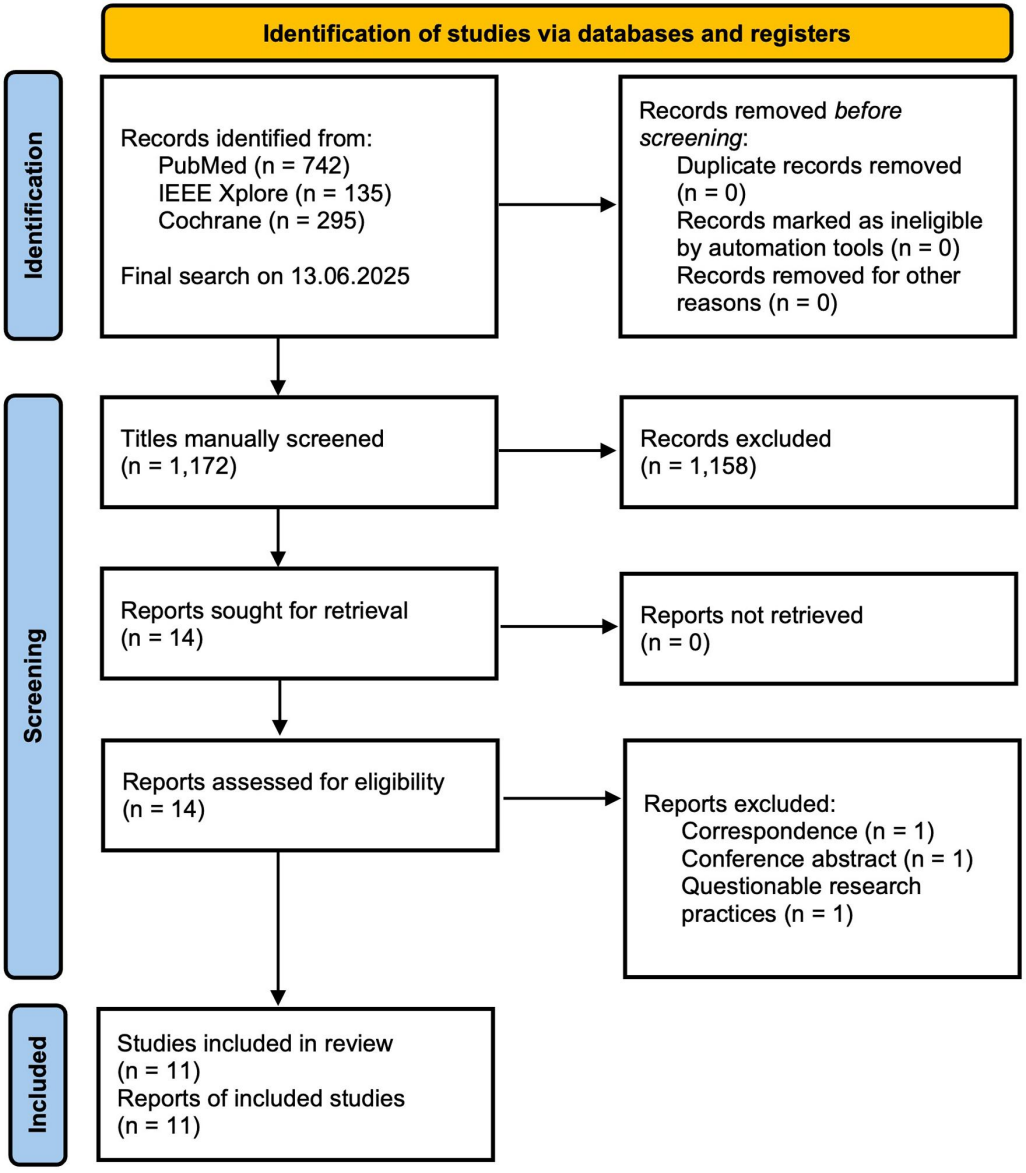
PRISMA flow diagram illustrating study selection. A list of the excluded studies (*n* = 3), their citations and corresponding rationales for exclusion can be found in the Supplementary Table S3. This flow diagram template is licensed under CC BY 4.0 [Source: Page et al. (37)]. To view a copy of this license, visit https://creativecommons.org/licenses/by/4.0/.

The remaining full-text articles were reviewed for eligibility. One study was excluded due to concerns about the integrity of its methodology and interpretation, including the overinterpretation of weak results (AUC = 0.53; Relative Erro*r* = 1.08) and a pattern of self-citation (38). This resulted in a final inclusion of *n* = 11 studies. A PRISMA Flow Diagram detailing the study selection process is depicted in Figure 1. A list of the excluded studies (*n* = 3), their citations and corresponding rationales for exclusion can be found in the Supplementary Table S3.

### 2.3 Data extraction

Data extraction was performed using a standardized form by a clinician-scientist trained in medicine and computer science, with domain expertise spanning both PDA and AI. Independent verification was performed by a second reviewer, with discrepancies resolved by consensus within the multidisciplinary author team including professors in neonatology, computer science, and pediatric cardiology. Extracted variables included study details, population characteristics, data sources, AI methods, evaluation metrics, validation strategies, performance results, key limitations, and information regarding model explainability and availability. A full summary of the extracted data is presented in the Supplementary Table S1.

### 2.4 Data synthesis and analysis

Given the small number of eligible studies (*n* = 11) and their substantial methodological heterogeneity, formal outcome assessments were not feasible. Instead, a narrative synthesis was conducted to compare and summarize the studies across key methodological and performance-related dimensions.

### 2.5 Risk of bias assessment

The risk of bias for diagnostic and predictive studies was evaluated using the Prediction model Risk Of Bias Assessment Tool (PROBAST) (39), while analytical cross-sectional studies were assessed using the Joanna Briggs Institute (JBI) Checklist (40). Reporting quality was assessed using the Minimum Information About Clinical Artificial Intelligence Modeling checklist (MI-CLAIM) (41).

### 2.6 Validation

Aspects of the review requiring interdisciplinary judgment, including conceptual design, evidence appraisal, narrative synthesis, and conclusions, were addressed collaboratively by a multidisciplinary author team with expertise in neonatology, computer science, and pediatric cardiology.

## 3 Results

A total of *n* = 11 studies were included in this review, exploring different aspects of PDA detection, diagnosis, risk stratification, or management using some form of AI. Studies varied in design, objectives, approaches, and validation strategies.

### 3.1 Aspect of PDA

The included studies addressed five key areas of PDA management: Diagnosis and Screening (33, 42–45), pharmacological Treatment Response Prediction (46, 47), Risk Factor Identification (34, 48), Subphenotype Analysis (49) and Treatment complication prediction (50).

### 3.2 Research objectives

The research objectives of the included studies are outlined in Table 2.

**Table 2:** Research objectives of included studies, grouped by the aspect of PDA management addressed, and sorted by year of publication.

Study	Aspect of PDA	Research objective
Na et al. (34)	Risk Factor Identification	To identify risk factors associated with symptomatic PDA and assess the feasibility and performance of AI models versus logistic regression for prediction.
Jura et al. (48)	Risk Factor Identification	To evaluate the influence of maternal pathologies, medications, and neonatal factors on PDA risk, and develop predictive models using logistic regression, chi-square tests, Random Forest, and XGBoost.
Gómez-Quintana et al. (42)	Diagnosis/Screening	To develop a ML framework for early screening and detection of PDA and congenital heart disease in neonates using phonocardiograms.
Lei et al. (33)	Diagnosis/Screening	To assess the feasibility and performance of a lightweight CNN (MobileNet-V2) for detecting PDA in neonatal echocardiograms for potential edge deployment.
Erno et al. (44)	Diagnosis/Screening	To create a DL model to classify echocardiographic video clips for PDA presence or absence and evaluate its performance.
Park et al. (43)	Diagnosis/Screening	To develop and assess a ML model and AI diagnostic support system for early PDA detection to improve accuracy and timeliness.
Chang et al. (45)	Diagnosis/Screening	To define radiographic features of severe PDA in chest x-rays of preterm infants using DL and ML methods and evaluate their predictive value.
Matsushita et al. (49)	Subphenotype Analysis	To apply unsupervised ML to identify subphenotypes of preterm infants with hemodynamically significant PDA for stratified intervention strategies.
Liu et al. (46)	Pharmacological Treatment Response Prediction	To develop and validate an interpretable ML model to predict NSAID efficacy in closing hemodynamically significant PDA in preterm infants under 30 weeks.
Sharma et al. (47)	Pharmacological Treatment Response Prediction	To train and validate a DL model to predict PDA closure likelihood after initial pharmacotherapy in preterm infants.
Zhang et al. (50)	Treatment Complication Prediction	To develop and compare four ML algorithms to identify the optimal model for predicting post-intervention platelet count decline in children with PDA.

PDA, Patent ductus arteriosus; AI, Artificial intelligence; XGBoost, Extreme Gradient Boosting; ML, Machine learning; CNN, Convolutional neural network; DL, Deep learning; NSAID, Nonsteroidal Anti-Inflammatory Drug.

### 3.3 Study design

Ten of the eleven studies reviewed were retrospective, with Gómez-Quintana et al. (42) being the only study that prospectively recruited participants. Most studies were conducted at single centres, except for Sharma et al. (47), which sourced data from two hospitals, and Na et al. (34), which utilized data from a nationwide registry encompassing 74 neonatal intensive care units. Gómez-Quintana et al. (42) technically involved two centers, though one of these contributed only three patients (1% of the total sample).

### 3.4 Definitions of study groups

While some studies differentiated between PDA and no PDA as confirmed by echocardiography (33, 42, 44, 48), others focused on distinguishing between symptomatic and asymptomatic PDA (34, 43, 45). However, there was significant heterogeneity in these definitions across studies. Na et al. (34) characterized symptomatic PDA based on clinical signs such as murmur, bounding pulse, hypotension, respiratory difficulty, pulmonary congestion, or cardiomegaly, with or without echocardiographic confirmation. In contrast, Park et al. (43) defined symptomatic PDA as infants who required pharmacological or surgical intervention within the first 15 days of life. Chang et al. (45) applied echocardiographic criteria, defining symptomatic PDA as a transductal diameter ≥1.5 mm and a left atrial-to-aortic diameter ratio ≥1.4:1.

Another term used to classify study groups was hemodynamically significant PDA (hsPDA). Matsushita et al. (49) studied hsPDA without a control group, defining it solely based on echocardiographic findings—specifically, an instance of PDA diameter >1.5 mm or an LA/Ao ratio >1.5 within the first two weeks of life. Liu et al. (46) used a combined definition that included both echocardiographic thresholds (PDA diameter ≥1.5 mm, LA/Ao ratio ≥1.4) and clinical indicators of systemic ischemia (e.g., hypotension, tachycardia, oliguria) and pulmonary overcirculation (e.g., continuous murmur, apnea, respiratory distress, increased oxygen or ventilation requirements).

In pharmacological treatment response prediction studies, definitions of treatment success varied. Liu et al. (46) defined success based on echocardiographic confirmation of PDA closure within 72 h after treatment, while Sharma et al. (47) defined successful closure as the absence of repeat pharmacotherapy, transcatheter occlusion, or surgical ligation before neonatal intensive care unit (NICU) discharge.

Zhang et al. (50), examining outcomes after transcatheter PDA closure, defined decline in platelet count (DPC) as a platelet count decline of ≥25% and NO-DPC as a decline of <25%.

### 3.5 Inclusion criteria

The inclusion criteria varied across studies. In retrospective studies, data availability was a primary consideration. For example, Erno et al. (44) and Lei et al. (33) included patients who had undergone echocardiography with a ductal view, while Chang et al. (45) required chest x-ray images taken within 4 days before or 3 days after an echocardiogram. Na et al. (34) focused on patients registered in the Korean Neonatal Network a nationwide neonatal registry, while Jura et al. included all NICU patients at their institution with complete maternal and neonatal records (48).

Seven of the eleven studies exclusively focused on preterm infants (gestational age < 37 weeks) (34, 43–47, 49). Among these, three studies further restricted their populations based on specific preterm categories: Matsushita et al. (49) examined extremely preterm births (≤28 weeks), Liu et al. (46) included very preterm births (≤30 weeks), and Na et al. (34) targeted very low birth weight preterm infants (<1,500 g).

In contrast, Gómez-Quintana et al. (42) included neonates independent of gestational age, with preterm infants comprising only 9% of the study participants, while Jura et al. (48) reported 69% preterm and 31% term participants. Lei et al. (33) did not report the gestational ages of their cohort.

Additional criteria were specified in some studies depending on their research objectives. For instance, Liu et al. (46) and Sharma et al. (47) included infants who received pharmacologic treatment for PDA to evaluate treatment response, while Zhang et al. (50) included children with PDA who underwent successful transcatheter closure to predict occurrence of post-interventional decline in platelet count. Gómez-Quintana et al. (42) focused on the development of a screening method and thus included only clinically asymptomatic newborns. Matsushita et al. (49), solely examining cases of hemodynamically significant PDA, incorporated this definition into their inclusion criteria.

The retrospective study periods also varied significantly, ranging from 1 year (44), to 3 years (48), 5 years (33, 34), 6 years (47), 7 years (46, 50), and up to 10 years (42, 43, 45, 49).

### 3.6 Exclusion criteria

Six studies excluded newborns with significant congenital heart disease (CHD) or pulmonary hypertension (33, 34, 44, 46, 47, 50). For instance, Sharma et al. (47) excluded participants exhibiting presence of other cardiac lesions (47), while Erno et al. (44) and Lei et al. (33) specifically excluded subjects with right-to-left PDA flow or complex CHD, except for patent foramen ovale (PFO), atrial septal defect (ASD), ventricular septal defect (VSD), or bicuspid aortic valve. Liu et al. (46) similarly excluded cases of complex CHD but allowed for ASD and small VSD. Na et al. (34) chose to exclude infants with any major congenital anomalies. Gómez-Quintana et al. (42), aiming to develop a screening method for PDA and other CHD, excluded infants with vivid clinical signs of CHD or pulmonary hypertension. Zhang et al. (50) excluded other CHD requiring surgery. In contrast, Chang et al. (45) deliberately chose not to exclude congenital heart or lung diseases to create a more clinically realistic study population, acknowledging the high rate of comorbidity between PDA and these conditions.

Death within the first three days of life was another frequent exclusion criterion. Park et al. (43) and Na et al. (34) excluded such cases because confirmation or evaluation of PDA would not be feasible. Matsushita et al. (49) excluded infants who died in the delivery room or were transferred to another institution before data collection.

In studies where group classification depended on treatment, additional exclusions were applied to ensure focus on relevant populations. Liu et al. (46) excluded infants with spontaneous PDA closure, those with drug contraindications requiring direct surgical ligation, and those with a history of NSAID treatment at other hospitals; Na et al. (34) excluded infants who received prophylactic or presymptomatic PDA treatment and Sharma et al. (47) excluded infants who received indomethacin for IVH prophylaxis. To avoid potential confounding effects on platelet count decline, Zhang et al. (50) also excluded patients with bleeding or hematologic disorders, preoperative heparin or chronic antiplatelet use, infective endocarditis or uncontrolled infections, and those with baseline platelet counts <100 × 10^9^/L.

Finally, missing or incomplete data led to exclusions in Na et al. (34), Liu et al. (46), Sharma et al. (47) and Jura et al. (48).

### 3.7 Sample size

After applying exclusion criteria, the study populations were generally small, with most studies including fewer than 500 participants: 66 (44), 113 (49), 174 (47), 182 (46), 201 (48), 265 (42), 300 (33), 330 (50) and 409 (43). Chang et al. (45) and Na et al. (34) were outliers with 4,617 and 8,369 participants respectively.

### 3.8 Study population characteristics

Eight of the eleven studies provided information on their study populations (34, 42, 43, 46–50). Lei et al. (33), Chang et al. (45), and Erno et al. (44), relying on imaging datasets, omitted detailed population data.

As highlighted by varying inclusion criteria, gestational age varied widely in study populations. Gómez-Quintana et al. (42) reported a median of 39 weeks (IQR: 38–40) for predominantly term infants, while Liu et al. (46), Matsushita et al. (49), Na et al. (34), and Park et al. (43) focused on preterm populations, with medians or means ranging from 26.3 weeks to 29.1 weeks.

Birth weight also differed significantly. Liu et al. (46) and Matsushita et al. (49) reported medians of 980 g (IQR: 860–1,160) and 715 g (IQR: 610–840), respectively, while Na et al. (34) and Park et al. (43) observed means of around 1,100 g.

Males outnumbered females in most studies (49.2%–66.7% male). Additional data reported in some studies included, among others, head circumference, cesarean births, multiple births, small for gestational age (SGA) status, Apgar scores, maternal factors, fetal anomalies, intraventricular hemorrhage (IVH) occurrence, and mortality. Notably, Matsushita et al. (49) reported a 49.6% mortality rate, likely attributable to their focus on preterm neonates with a gestational age of ≤28 weeks.

### 3.9 Data modality

In terms of input data, two main trends can be noted. Six of eleven studies focused on a multidimensional collection of patient data, ranging in number from six to one hundred eight different variables (34, 43, 46, 48–50). These variables were categorized into distinct subgroups dependent on the focus of each study, including demographic factors, maternal factors, prenatal factors, delivery and post-birth factors, postnatal clinical factors, blood pressure-related features, laboratory parameters, pre- and intra-operative factors and echocardiographic measurements. Liu et al. (46) and Park et al. (43) included a balanced number of pre- and postnatal factors, whereas Na et al. (34) included primarily prenatal and delivery factors, and Matsushita et al. (49) focused solely on postnatal data collected within the first three days of life.

The remaining five studies focused on high-dimensional unimodal data, including phonocardiogram recordings (42), echocardiography video frames (33, 34, 47), and chest x-rays (45). Two of these studies, Chang et al. (45) and Sharma et al. (47), pursued multimodal approaches, combining imaging data with perinatal variables.

All three studies using echocardiograms utilized the ductal view (high left parasternal sagittal), with Erno et al. (44) choosing to work with color Doppler only and Lei et al. (33) using frames with and without color Doppler. Sharma et al. (47) was the only echocardiogram study that reported results of experiments with other views.

### 3.10 AI methodology

Six of eleven studies focused on a single ML method. The five studies which explored more than one method varied in approach. Jura et al. (48), Zhang et al. (50) and Na et al. (34) compared two, four and five different ML methods for their prediction models, respectively. Sharma et al. (47) compared imaging-only and multimodal convolutional neural networks with random forest and logistic regression models based on perinatal data alone. Chang et al. (45) initially employed convolutional neural networks (CNNs) to perform feature extraction and subsequently leveraged XGBoost to exploit radiographic and clinical features in an explainable way.

Traditional ML methods were employed more often (nine of eleven studies) than DL methods (five of eleven studies). All studies but one used supervised ML methods with labeled data. The exception, Matsushita et al. (49), clustered unlabeled data using agglomerative hierarchical clustering with the Euclidean distance measure and the Ward method, which minimizes intra-cluster variance, on principal components. An overview of the machine learning approaches observed in the included studies is shown in Table 3.

**Table 3:** Overview of machine learning approaches in the included studies, categorized by learning type, model type, models architectures, frequency, and cited studies.

Category	Subcategory	Models	*n*	Studies
Supervised	Traditional ML	RF, XGBoost, LightGBM, SVM, k-NN	8	Liu el al. (46), Na el al. (34), Park el al. (43), Gómez-Quintana el al. (42), Chang el al. (45), Jura el al. (48), Sharma el al. (47), Zhang el al. (50)
	DL	ResNet-50 CNN, MobileNet-V2, CNN, ConvNeXt CNN, EchoNet-Pediatric (51) CNN, MLP	5	Na el al. (34), Lei el al. (33), Erno el al. (44), Chang el al. (45), Sharma el al. (47)
Unsupervised	Traditional ML	Agglomerative Clustering	1	Matsushita el al. (49)
	DL	None	0	None

*n*, Frequency; ML, Machine learning; DL, Deep learning; RF, Random forest; XGBoost, Extreme Gradient Boosting; LightGBM, Light Gradient Boosting Machine; SVM, Support Vector Machine; k-NN, k-Nearest Neighbors; CNN, Convolutional neural network; MLP, Multilayer Perceptron.

Among the supervised models, the most common were decision tree-based ensemble methods, used in eight of ten studies. These included random forest (34, 43, 46–48, 50), XGBoost (gradient boosted decision trees) (42, 45, 48, 50), and Light Gradient Boosting Machine (L-GBM) (34). The next most common supervised method, present in five of ten studies, was the neural network. This included convolutional neural networks of varying architectures [ResNet-50 (44), ConvNeXt (45), MobileNet-V2 (33), EchoNet-Pediatric (47, 51)] and an instance of a multilayer perceptron (MLP) (34). Erno et al. (44) and Lei et al. (33) leveraged pretrained ImageNet weights, while Sharma et al. (47) utilized transfer learning via a pretrained EchoNet-Pediatric (51) backbone. Data augmentation to compensate for class imbalance was reported only by Lei et al. (33). Support vector machine (SVM) and instance-based learning with k-nearest neighbors (k-NN) were each employed in only one study (34).

### 3.11 Experiments for model choice/ablation studies

Supplementary methodologies aimed at achieving the most effective model solutions were utilized across all studies and can be categorized into four key areas: feature engineering and optimization, model development and training, data selection and input configuration, and output aggregation and thresholding.

In feature engineering and optimization, Gómez-Quintana et al. (42) utilized feature aggregation to improve classification, while ablation studies identified optimal subsets, such as the Top-15 features for PDA vs. healthy cases. Similarly, Park et al. (43) compared the outputs of four models to determine the most effective feature sets. Matsushita et al. (49) applied principal component analysis (PCA) to reduce dimensionality, address collinearity, and enhance clustering, alongside normalization and imputation to handle missing values. Liu et al. (46) used binary logistic regression to identify factors influencing treatment efficacy and Zhang et al. (50) applied a two-step process involving univariate analysis followed by extra-trees for feature selection, using AUC comparisons to evaluate model performance.

For model development and training, Gómez-Quintana et al. (42) applied regularization techniques such as subsampling and shrinkage (learning rate) to control overfitting. Key hyperparameters, including tree depth, subsample ratios, feature selection ratios, and the number of decision trees, were tuned for optimal performance. Similarly, Na et al. (34) and Jura et al. (48) used grid searches and stratified five-fold cross-validation to optimize hyperparameters. Sharma et al. (47) compared three vision-based architectures—a scratch-built 3D CNN and two pretrained models (Swin3D and EchoNet-Pediatric)—ultimately selecting EchoNet-Pediatric (51) as the backbone for both an imaging-only CNN and a multimodal model incorporating echocardiographic clips and perinatal data.

In the area of data selection and input configuration, Chang et al. (45) tested rule-based filtering and a novel “human-guided easy learning” approach to curate datasets, with the latter improving performance. In this method, a medical professional was first trained by reviewing 173 chest x-ray (CXR) images—balanced between positive and negative sPDA cases—and received the correct diagnosis after each review. After this training, the professional evaluated 4,654 additional images, and only the 3,349 images that were correctly classified were used to train the model, based on the assumption that these clearer cases contain more distinct and anatomically relevant features of sPDA. Additionally, Chang et al. (45) evaluated various input types—raw images, heart-segmented masks, and thorax-segmented masks—with combined inputs yielding the best results. Sharma et al. (47) evaluated EchoNet-Pediatric (51) based models across echo view subsets (non-PDA, 2D PDA, Color PDA, Color Compare PDA, and Overall) and benchmarked against perinatal-only logistic regression and random forest models.

Finally, comparisons of output aggregation and thresholding were explored. Lei et al. (33) focused on optimizing classification thresholds using frame- and clip-level criteria, while Erno et al. (44) explored predictions at both clip- and study-levels.

### 3.12 Performance metrics reported

Across the studies, performance metrics varied. The AUC was the most commonly reported metric, appearing in ten studies (33, 34, 42, 44–48, 50), often accompanied by accuracy, sensitivity, and specificity (33, 34, 44, 45, 47, 48). Precision, recall, and false negative rate were specific to Park et al.'s (43) evaluation, while Lei et al. (33) and Sharma et al. (47) uniquely reported positive and negative predictive values (PPV, NPV). Matsushita et al. (49), focusing on unsupervised clustering without ground truth, did not provide conventional performance metrics.

Nine studies calculated metrics on a separate test set distinct from the training and validation data (33, 34, 43–48, 50). Liu et al. (46) and Chang et al. (45) used 10% of the data for testing, Erno et al. (44) and Lei et al. (33) approximately 15%, Na et al. (34) and Park et al. (43) 20%, while Sharma et al. (47), Jura et al. (48), and Zhang et al. (50) each allocated 30%. In contrast, Gómez-Quintana et al. (42) did not use an independent test set, instead relying on outer 10-fold cross-validation to derive performance metrics. Matsushita et al. (49), employing an unsupervised clustering approach, did not necessitate data splitting.

### 3.13 External validation

Ten of eleven studies (33, 34, 42–45, 47–50) did not perform any external validation. Liu et al. (46) conducted limited external validation using data from only two patients at a second hospital.

### 3.14 Results/performance

Reported model performance should be interpreted with caution, as differences in study design limit direct comparability and the presence of methodological bias may contribute to overfitting with artificially inflated performance metrics.

#### 3.14.1 Diagnosis and screening

The performance metrics of the best performing models in the category diagnosis and screening are depicted in Table 4.

**Table 4:** Performance metrics from the best-performing models in PDA diagnosis and screening studies. Metrics are not directly comparable due to variations in study design. Given the high risk of bias, overfitting with artificial inflation of performance metrics is likely.

Study	Model	AUC	Sensitivity	Specificity	PROBAST risk of bias
Erno et al. (44)	USVN CNN model (study-level prediction)	0.93	0.83	0.89	High
Lei et al. (33)	MobileNet-V2 CNN model	0.88	0.76	0.87	High
Gómez-Quintana et al. (42)	XGBoost PDA detection model	0.761	Not reported	Not reported	High
Chang et al. (45)	XGBoost model (ratio features + clinical data)	0.74	0.42	0.94	High
Study	Model	Accuracy	Precision	Recall	
Park et al. (43)	Perinatal RF model	82%	38%	76%	High

USVN, Ultrasound Video Network; CNN, Convolutional neural network; XGBoost, Extreme Gradient Boosting; PDA, Patent ductus arteriosus; RF, Random forest.

Most studies achieved reasonable AUC values, demonstrating feasibility for PDA detection. Erno et al.'s (44) study-level predictions reported an AUC of 0.93 (95% CI: 0.89–0.98), sensitivity of 0.83, and specificity of 0.89. Clip-level predictions achieved an AUC of 0.86 (95% CI: 0.83–0.90). Lei et al. (33) reported an AUC of 0.88, sensitivity of 0.76, specificity of 0.87, positive predictive value of 0.84, and negative predictive value of 0.80. Each test video clip was processed in approximately seven seconds on an Intel 8-core i7 processor without Graphics Processing Unit. Gómez-Quintana et al. (42) reported a validation AUC of 0.76 for PDA detection and 0.77 for other congenital heart disease, with similar test AUCs of 0.74 and 0.78. These results were derived from cross-validation without an independent test set. Chang et al.'s (45) XGBoost models achieved AUCs ranging from 0.72 to 0.75, with specificity ranging from 0.91 to 0.95 and sensitivity from 0.33 to 0.42. The highest AUC of 0.75 was achieved by the model trained using feature vectors from the CNN and clinical data, with specificity of 0.95 and sensitivity of 0.33. The best balance between specificity and sensitivity was achieved by the model trained on extracted ratio features and clinical data, with an AUC of 0.74, specificity of 0.94, and sensitivity of 0.42. Park et al.'s (43) models reported accuracy between 71% and 84%, precision between 24% and 42%, and recall between 61% and 76%. Whilst Model_perinatal + bp achieved the best accuracy and precision at 84% and 42% respectively, Model_perinatal achieved the best balance of accuracy (82%), precision (38%), and recall (76%). The AI-based diagnostic support system, employing Model_perinatal, improved NICU professionals’ performance, with accuracy increasing from 48% to 76%, precision from 46% to 80%, and recall from 52% to 73%. Diagnoses were made 2.5 (nurses and doctors) and 3.1 days (doctors only) earlier with AI compared to 2.1 and 3 days earlier without AI.

#### 3.14.2 Pharmacological treatment response prediction

Liu et al. (46) reported a 45.6% pharmacological PDA closure rate with a predictive model achieving an AUC of 0.792 (95% CI: 0.457–0.841). Sharma et al. (47) observed a 60% success rate, using the absence of further PDA interventions as a surrogate outcome. In their model comparisons, an EchoNet-Pediatric (51) backbone outperformed a scratch-built 3D CNN and pretrained Swin3D for imaging-only prediction. The multimodal model, combining imaging with perinatal data, achieved superior performance (AUC: 0.82, F1: 0.78, sensitivity: 0.76, specificity: 0.70, PPV: 0.79, NPV: 0.66), outperforming both the imaging-only model (AUC: 0.66) and baseline models using perinatal data alone (logistic regression: AUC 0.66; random forest: AUC 0.74).

#### 3.14.3 Risk factor identification

Both risk factor identification studies showed reasonable predictive performance with AUCs of 0.82 (34) and 0.87 (48).

Na et al. (34) identified gestational age, invasive mechanical ventilation, sepsis, and birth weight as key features for symptomatic PDA (sPDA) prediction and sepsis, supplemental oxygen at birth, noninvasive ventilation, and birth temperature as key features for treatment necessity prediction. In their study comparing 6 ML methods, ensemble models such as Random Forest and Light Gradient Boosting Machine showed modest performance improvements over Multiple Logistic Regression. Light Gradient Boosting Machine achieved an accuracy of 0.77 and an AUC of 0.82, while Multiple Logistic Regression achieved an accuracy of 0.76 and an AUC of 0.81. Multiple Logistic Regression reported a sensitivity of 0.85, compared to 0.65 for Light Gradient Boosting Machine and 0.64 for Random Forest. Light Gradient Boosting Machine reported a specificity of 0.84, compared to 0.83 for Random Forest and 0.60 for Multiple Logistic Regression. For predicting sPDA requiring treatment, all models achieved an accuracy of approximately 0.85. Random Forest reported a sensitivity of 0.97 and specificity of 0.36, while Multiple Logistic Regression reported a sensitivity of 0.85 and specificity of 0.98, although this may be a typographical error (reported confidence interval of 0.28–0.32).

Jura et al. (48) identified prolonged rupture of membranes (PROM) as a strong predictor of PDA (OR: 13.03, *p* < 0.001), with lower gestational age (OR: 0.85, *p* = 0.042) and lower birth weight (OR: 0.72, *p* = 0.029) also showing significant associations (*p* < 0.05). In predictive modeling, XGBoost outperformed Random Forest with 81.4% accuracy, 92.5% sensitivity, 57.9% specificity, and an AUC of 0.872. Random Forest achieved 76.3% accuracy, 47.4% sensitivity, and 90% specificity.

#### 3.14.4 Subphenotype analysis

Matsushita et al. (49) identified two clusters. The inflamed cluster had leukocyte counts of 10.6 × 10^3^/uL compared to 5.97 × 10^3^/uL, neutrophil percentages of 69.7% compared to 46%, neutrophil-to-lymphocyte ratios of 3.23 compared to 1.14, mean corpuscular volume of 108.5 fL compared to 115.6 fL, and mean corpuscular hemoglobin concentration of 34.9 g/dL compared to 33 g/dL (*p* < 0.001 for all comparisons). The respiratory acidosis cluster had pH levels of 7.23 compared to 7.28 (*p* = 0.005) and pCO2 levels of 45.5 mmHg compared to 38.3 mmHg (*p* < 0.001). No differences were observed in demographics, IVH severity, SGA prevalence, or twin births.

#### 3.14.5 Treatment complication prediction

Zhang et al. (50) identified six key predictors of post-intervention decline in platelet count in children with PDA through univariate analysis and extra-trees feature selection: systolic pulmonary artery pressure, pulmonary valve velocity, age, weight, defect size, and mean pulmonary artery pressure. Among the models based on these predictors, Random Forest achieved the highest performance with a Train-AUC of 0.81 and Test-AUC of 0.71. Logistic Regression, AdaBoost, and XGBoost yielded Train-AUCs of 0.68, 0.71, and 0.70, respectively.

### 3.15 Model examination/explainability

Model explanation and explainability methods varied across studies, ranging from thorough techniques to limited or absent approaches.

Several studies focused on statistical feature importance. Jura et al. (48) used logistic regression and chi-squared tests to rank predictive features. Matsushita et al. (49) validated patterns identified during hierarchical clustering using statistical tests, including Mann–Whitney U and chi-square tests. Liu et al. (46) employed variable importance plots to rank plasma albumin level, platelet count, and 24 h urine volume as the top predictors for PDA closure success, with marginal effect plots revealing nonlinear relationships.

Na et al. (34) used Shapley Additive Explanations (SHAP)—a method that quantifies how much each input feature contributes to a model's prediction—to highlight gestational age, invasive mechanical ventilation, and birth weight as key predictors of symptomatic PDA. Similarly, Zhang et al. (50) used SHAP to show that higher systolic PAP, larger defect size, younger age, lower weight, and increased pulmonary valve velocity were associated with elevated risk of post-interventional platelet count decline. Chang et al. (45) also applied SHAP, identifying radiographic features such as the cardiothoracic ratio as influential in their XGBoost model.

Visualization techniques were present in three imaging-based studies. Chang et al. (45) used GradCAM++ to identify thoracic regions associated with PDA-related changes, such as cardiomegaly and lung opacity, aiding the validation of radiographic predictors like the cardiothoracic ratio for symptomatic PDA. Sharma et al. (47) generated saliency maps to highlight image regions relevant to predictions, publishing one effective and ineffective example. Erno et al. (44), provided a single figure visualizing frame-level attention changes during one echocardiogram video. Lei et al. (33) did not report any explainability methods.

Park et al. (43) demonstrated the only study focused on clinical usability, developing an AI-based diagnostic support system featuring interactive dashboards. These dashboards included components for visualizing long-term electronic health record trends, comparing clinical patterns, and integrating classification probabilities with symptom development predictions.

### 3.16 Fairness

None of the examined studies explicitly mentioned aspects of AI fairness, such as addressing biases in training data or ensuring equitable performance across demographic groups in their publications.

### 3.17 Risk of bias

The risk of bias across included studies was assessed using the Prediction Model Risk of Bias Assessment Tool (PROBAST) (39), summarized in Table 5 and available in more detail in the Supplementary Material. The overall risk of bias across studies was high, with most concerns concentrated in the analysis domain. Common issues included small sample sizes leading to low events-per-predictor ratios, which increased susceptibility to overfitting—particularly in high-dimensional models. Many studies selectively reported performance metrics, often limiting evaluation to AUC alone, and employed inadequate internal validation strategies without any attempt at external validation. Additionally, missing data were frequently handled suboptimally, either through case exclusion without imputation or the via simplistic imputation methods.

**Table 5:** A summary of the results of the risk of bias assessment performed using the Prediction Model Risk of Bias Assessment Tool (PROBAST) (39), comprising four assessment categories: participants, predictors, outcome and analysis. These results are available in more detail in Supplementary Table S2.

PROBAST assessment category	Study									
	Gómez-Quintana et al. (42)	Liu et al. (46)	Na et al. (34)	Park et al. (43)	Erno et al. (44)	Chang et al. (45)	Lei et al. (33)	Jura et al. (48)	Sharma et al. (47)	Zhang et al. (50)
Participants	Low	Unclear	Unclear	Low	Unclear	Low	Unclear	Unclear	Unclear	Unclear
Predictors	Low	Low	Low	Low	Low	Low	High	Low	Low	Low
Outcome	Low	Low	Unclear	High	Low	Low	Low	Unclear	High	Unclear
Analysis	High	High	Low	High	High	High	High	High	High	High
Overall Risk of Bias	High	High	Unclear	High	High	High	High	High	High	High

As the study by Matsushita et al. (49) did not involve a predictive model, risk of bias was instead evaluated using the Joanna Briggs Institute (JBI) Checklist (40). In this instance, while most criteria were met, two items remained unclear: 1) the use of objective, standard criteria for measuring the condition, as echocardiography—though considered the gold standard—lacks standardized definitions for parameters and cut-offs; and 2) the handling of confounding variables, due to the absence of explicit discussion on potential confounders and the lack of a matched control group.

### 3.18 Minimum information about clinical artificial intelligence modeling (MI-CLAIM)

While reporting quality was generally sound, several checklist items were consistently unaddressed. Clinical utility metrics, such as positive predictive value (PPV) were only reported by Lei et al. (33) and Sharma et al. (47) and no study displayed adequate proof of reliability under data distribution shifts via external validation. Additionally, significant variability was observed in baseline comparisons and reproducibility practices.

#### 3.18.1 Comparison with baseline

Five of eleven studies compared their models to some form of baseline method (34, 42, 43, 47, 48). Gómez-Quintana et al. (42) compared their Top-15 features model to a trained neonatologist analyzing heart sound recordings, with the model outperforming the neonatologist in sensitivity (0.72 vs. 0.62 at fixed specificity) and specificity (0.82 vs. 0.71 at fixed sensitivity). Na et al. (34) used multiple logistic regression as the baseline, enabling direct comparisons with ML methods, while Park et al. (43) evaluated the performance of neonatal intensive care unit professionals with and without AI support. Sharma et al. (47) used random forest and logistic regression models trained on perinatal data alone as baselines for evaluating the added value of multimodal and imaging-only CNN models. Similarly, Jura et al. (48) employed logistic regression and chi-squared tests as traditional statistical baselines, with random forest also serving as a point of comparison for XGBoost.

#### 3.18.2 Public availability of data, models, or code

The availability of data, models, or code varied across studies, with most citing privacy concerns or omitting details on accessibility. Matsushita et al. (49) was the only study offering a publicly accessible resource, sharing code at https://github.com/fymatsushita/PDA, though no data were made available. Gómez-Quintana et al. (42) withheld data for privacy reasons but planned to deploy their models as a cloud-based tool at https://www.hearttone.org; however, the website was listed as “not found” at the time of this review. Chang et al. (45) and Erno et al. (44) stated that anonymized data could be requested but were not publicly available due to privacy restrictions, and neither provided code or models. Na et al. (34) similarly referenced confidentiality policies from the Korean Neonatal Network, restricting data access to approved research activities, with no mention of public code or models. The remaining studies did not report on the availability of data, models, or code.

## 4 Discussion

This review highlights the emerging but still limited role of artificial intelligence in the context of PDA.

PDA represents a uniquely complex clinical challenge: its hemodynamic impact lies on a continuum, evolves dynamically over time, and predominantly affects a highly vulnerable patient population often burdened with multiple comorbidities. These factors are further complicated by the absence of standardized subgroup definitions, such as “symptomatic” or “hemodynamically significant” PDA, as well as the lack of consensus regarding which patients benefit from which interventions. As a result, management relies on institution specific standards and case-by-case expert appraisal, limiting consistency in care and disadvantaging those in underserviced areas. These factors have led to a fragmented research landscape, where study designs, outcome measures, and clinical relevance are difficult to compare. Against this backdrop, AI holds significant potential to support more consistent, data-driven decision-making by processing high-dimensional clinical data and capturing subtle, temporal patterns beyond human capability. The promise of AI in this setting lies not in replacing clinicians, but in augmenting their ability to interpret complex information and tailor management more precisely to the individual patient. Despite growing interest and demonstrated feasibility across a range of applications, current research remains constrained by methodological, clinical, and ethical limitations. The following discussion critically evaluates these challenges, synthesizes common themes across the included studies, and outlines key directions for future research and clinical translation.

Given the novelty of AI for PDA and the subsequent lack of precedent, investigators of included studies were forced to make methodological decisions within an uncharted research landscape. Despite these obstacles, authors not only showcased feasibility but also made commendable efforts in areas such as model interpretability, edge deployment, and clinical integration. Nevertheless, the studies included in this review are not without their limitations, including high risk of bias, heterogeneous non-standardized study group definitions, issues with generalizability, methodological challenges in machine learning approaches, gaps in model evaluation and validation, insufficient focus on explainability, clinical utility and fairness, and limitations in transparency and reproducibility.

### 4.1 Performance metrics do not equate clinical applicability

Although many of the included studies report AUCs in the moderate-to-high range, these results must be interpreted with caution and should not be mistaken for evidence of clinical applicability. As highlighted by the PROBAST evaluation, all but one study [Na et al. (34); risk of bias ‘unclear’] exhibit a high risk of bias, meaning that seemingly strong model performance rests on methodologically fragile foundations: most are developed from retrospective, single-center datasets, rely on limited event numbers relative to model complexity, and all lack adequate external validation. Under such conditions, high AUCs likely reflect overfitting to idiosyncrasies of the development dataset, inflating internal performance, rather than genuine clinical signal.

Gómez-Quintana et al. (42) derived their AUC exclusively from cross-validation without an independent test set, Erno et al. (44) tested performance on only 11 patients, while others relied on surrogate study group and outcome definitions, such as treatment initiation (43) or the absence of subsequent intervention (47), that do not necessarily represent true ductal physiology or clinically meaningful endpoints.

Na et al. (34) was the only analysis not deemed high risk of bias according to PROBAST. While their Light Gradient Boosting Machine model showed reasonable performance (AUC 0.82; sensitivity 0.65; specificity 0.84), the model's target outcome warrants scrutiny. “Symptomatic PDA” was anchored to treatment necessity rather than objective clinical or echocardiographic criteria, rendering it a surrogate of limited validity—particularly in the context of a retrospective 5-year cohort during which PDA management strategies shifted substantially from early targeted treatment toward more conservative, expectant approaches.

Furthermore, closer examination of the reported metrics—such as diagnostic models achieving high specificity but very low sensitivity (45), treatment response models reporting moderate discrimination but extremely wide confidence intervals (46), or PDA detection models with 24%–42% precision (43)—highlight that superficially moderate to high performing models are not necessarily generalizable or aligned with clinically meaningful decision thresholds, where missed diagnoses or misclassified treatment candidates carry significant consequences.

The absence of external validation and prospective assessment precludes any meaningful appraisal of model performance in heterogeneous real-world settings, which significantly affect model behavior.

For AI to influence PDA care in a reliable and ethically defensible manner, future studies must adopt larger populations, multicenter designs; employ standardized study group and outcome definitions; incorporate robust internal and external validation frameworks with subsequent prospective evaluation; and demonstrate value beyond established clinical assessment pathways. Only through such methodology can performance metrics be translated into clinically applicable and beneficial AI tools.

### 4.2 Heterogeneous study group definitions

Significant heterogeneity in diagnostic criteria for PDA and its hemodynamically significant or symptomatic subgroups was observed across the studies included in this review, leading to inconsistent study groups. For example, Na et al. relied on clinical signs for PDA diagnosis without echocardiographic confirmation and Park et al. (43) defined 'symptomatic’ PDA groups based on treatment decisions rather than symptoms. Matsushita et al. (49)'s use of static measures like worst diameter and LA/Ao ratios overlooked longitudinal hemodynamic changes important for guiding treatment. This variability reflects the ongoing lack of consensus that challenges PDA research and clinical practice more broadly, impeding both the comparability of results and their translation into clinical care. Furthermore, the exclusion of neonates with other congenital heart disease or pulmonary hypertension created artificially clean datasets that fail to represent the clinical reality of high comorbidity rates (33, 34, 42, 44, 46, 47, 50). Chang et al. (45) was the exception, better accounting for such complexities. While Liu et al. (46) utilized echocardiographic confirmation, Sharma et al. (47) defined successful ductal closure based solely on the absence of further treatment, rendering the treatment response prediction models incomparable and raising concerns about possible misclassification. These limitations hinder the interpretability, comparability, and clinical utility of the included studies, underscoring the general need for standardized criteria defining hemodynamically significant or symptomatic subgroups.

### 4.3 Generalizability

The generalizability of findings was constrained by the single-center design of all but three studies (34, 42, 47). In Gómez-Quintana et al. (42), one of two hospitals contributed only three patients, rendering Na et al. (34) and Sharma et al. (47) the only valid examples of multi-center design. External validation was rare, with Liu et al. (46) offering the sole attempt, albeit on just two patients, rendering results effectively non-generalizable. Small study populations and event rates were another limitation, with the exception of Na et al. (34) and Chang et al. (45). Imaging-focused studies (33, 44, 45), except for Sharma et al. (47), compounded these issues by omitting population demographics, likely due to the resource-intensive process of matching imaging data with clinical records. The reliance on retrospective datasets in all studies except Gómez-Quintana et al. (42) introduced additional challenges to generalizability. These datasets spanned 1–10 years, during which advances in technology and shifts in treatment practices likely affected data consistency and relevance. Na et al.'s (34) dataset, while large, lacked PDA-specific clinical details, and Matsushita et al.'s (49) use of averaged laboratory values may have obscured clinically relevant fluctuations. These challenges underscore the need for multicenter, prospectively designed studies with diverse, well-characterized populations and external validation.

### 4.4 Methodological challenges in machine learning approaches

Traditional ML methods dominated (nine of eleven studies), often reflecting constraints of small datasets, while DL approaches and modern state-of-the-art architectures were underutilized. Chang et al. (45) and Lei et al. (33) made this decision consciously, prioritizing explainability and edge deployment, respectively. Unsupervised ML, valuable for discovering patterns in unlabeled data, particularly helpful in the medical domain where data sharing and expert knowledge limit available labeled datasets, was employed only by Matsushita et al. (49). Echocardiography studies utilized data from single time points, missing the insights of serial appraisal. Lei (33) and Erno et al. (44) failed to capture temporal dynamics, relying on clip-level thresholds of frame-independent analysis, restricting their ability to detect dynamic features relevant to PDA. Sharma et al. (47) did not explore a perinatal-only CNN model, which could have provided a fairer benchmark for comparison against their proposed multimodal CNN, potentially overestimating the added value of multimodality. Manual preprocessing steps, such as phonocardiogram segmentation (42) and frame filtering (33), introduced labor-intensive processes that reduced scalability and potentially biased training. Overfitting was evident in non-ensemble models (34), while convolutional neural network architectures were employed where newer methods, such as Vision Transformers, may have improved performance (33, 44). Techniques such as data augmentation and transfer learning were employed in only one (33) and three studies (33, 44, 47) respectively. The lack of self-supervised pre-training, a promising method for leveraging unlabeled data, was another missed opportunity. Future research should adopt modern, scalable methodologies that balance interpretability with performance.

### 4.5 Model evaluation and validation gaps

Model evaluation and validation exhibited significant gaps, and overall model performance was generally modest, with the exception of echocardiography-based diagnostic prediction studies by Erno et al. (44) and Lei et al. (33). However, due to substantial variations in study design—including differences in inclusion and exclusion criteria, study populations, and methodologies—along with high risk of bias, performance metrics are likely inflated, are not directly comparable and must be interpreted within the context of each study. For example, the highest-performing diagnostic model by Erno et al. (44) was evaluated on a sample of just eleven patients, limiting confidence in its generalizability. Gómez-Quintana et al. (42) relied on internal cross-validation without a withheld test set and reported only the AUC, omitting metrics such as sensitivity and specificity. Chang et al. (45) demonstrated poor sensitivity, while Park et al.'s (43) model exhibited low precision (24%–42%), likely attributable to class imbalance; their additional reporting of the false negative rate (the inverse of recall) added little interpretative value. Jura et al. (48) failed to report the number of patients with and without PDA, making it impossible to evaluate class balance or contextualize sensitivity and specificity. Their reported odds ratios showed wide confidence intervals for some univariate predictors [e.g., PROM OR 13.03 [1.72–98.7], SARS-CoV-2 infection OR 2.44 [0.28–21.3]], reflecting low event counts. Similarly, Liu et al.'s (46) wide AUC confidence interval (0.457–0.841) underscored considerable variability and uncertainty in their model's performance. Finally, external validation was either absent or insufficient across all studies. To enhance reliability and generalizability, incorporating independent test sets, carefully selecting metrics, interpreting performance metrics appropriately, and conducting external validation are essential steps forward.

### 4.6 Explainability and clinical utility

Explainability, while addressed to some extent in most studies, exhibited notable limitations. Some imaging studies incorporated visualization methods, including saliency mapping and attention or activation heatmaps, though these were often demonstrated using only single or illustrative examples. While few studies applied more advanced techniques like SHAP or GradCAM++, others relied solely on global feature importance, which may not translate to individual patient explanations necessary for clinical implementation. Furthermore, explainability methods were not evaluated within the context of clinical workflows, raising uncertainty about their practical utility in supporting accurate clinical decisions.

Clinical utility was also constrained. Many studies employed artificially clean study populations that excluded common comorbidities, thereby failing to reflect the complexity of real-world PDA management. In addition, modest model performance with high risk of bias and a lack of external validation further undermined the clinical applicability of the proposed tools. For example, Park et al. (43) reported only a marginal improvement in diagnostic timing—0.1 days—when using their AI-assisted diagnostic workflow compared to clinician performance without AI. Moreover, many studies focused narrowly on binary classification tasks without accounting for disease severity, which reduces the clinical relevance of their outputs. Although echocardiography-based diagnosis prediction models (33, 44) achieved high classification accuracy, they concentrated on identifying PDA presence in preselected ductal views with color Doppler—a task that is typically straightforward for clinicians and offers limited value. The more pressing challenge lies in performing comprehensive severity appraisal across multiple full echocardiographic exams and the prediction of clinically relevant outcomes—areas where AI could provide meaningful support, as demonstrated by Sharma et al. (47). Similarly, Gómez-Quintana et al.'s (42) model classified PDA but failed to specify severity or co-occurring congenital heart disease (CHD), limiting its clinical benefit. Regarding integration into clinical practice, whilst Lei et al. (33) opted for a lightweight architecture to accommodate future edge deployment, Park et al. (43) was the only study to present a user interface for real-time model deployment. Moving forward, future research should focus on developing interpretable models that address clinically meaningful tasks and demonstrate tangible benefits at the point of care.

### 4.7 Fairness

The absence of explicit consideration of AI fairness in the examined studies highlights a critical gap in current research. Addressing biases in training data and ensuring equitable performance across demographic groups is essential for developing inclusive and clinically applicable artificial intelligence models.

### 4.8 Transparency and reproducibility

Transparency and reproducibility were major shortcomings. None of the studies provided publicly available datasets, reflecting a significant challenge when working with sensitive patient data, and only Matsushita et al. (49) made code accessible. Manual preprocessing steps, such as frame filtering (33) and phonocardiogram segmentation (42), added further barriers to reproducibility. Sharma et al. (47) did not report essential details regarding the number of frames per clip, subsampling strategies, or how multi-view data were processed, limiting transparency and hindering reproducibility. Addressing these gaps through the sharing of anonymized data, models, and code is an important step toward advancing research and fostering progress in this domain.

### 4.9 Limitations of the review process

Despite a comprehensive search across multiple databases, only eleven studies met the inclusion criteria. This small sample size, whilst reflecting the emerging nature of the field, limits the generalizability of the findings and the strength of the conclusions that can be drawn. The review was restricted to peer-reviewed articles published in English, excluding non-English publications and grey literature, which may have omitted relevant studies and introduced potential language and publication bias. The included studies exhibited significant heterogeneity in methodologies, data sources, population characteristics, and performance metrics, which precluded formal meta-analysis or quantitative synthesis. Key aspects requiring interdisciplinary judgment—including review conceptualization, interpretation of the evidence, narrative synthesis, and formulation of conclusions—were addressed by consensus within a multidisciplinary author team comprising experts in neonatology, computer science, and pediatric cardiology. However, data extraction was performed by a single reviewer, which may have introduced subjective interpretation or oversight; this risk was partially mitigated through independent verification by a second reviewer and consensus-based resolution of discrepancies within the author team. While the Prediction Model Risk of Bias Assessment Tool (PROBAST) (39) and Joanna Briggs Institute (JBI) tools (40) were used to assess risk of bias, their traditional design for conventional prediction models and cross-sectional studies does not fully align with the diverse and complex methodologies of AI-based clinical research, limiting their suitability for the included studies. Finally, the reliance on narrative synthesis, although necessary given the heterogeneity of the studies, is inherently less objective and reproducible compared to quantitative meta-analyses.

### 4.10 Implications for practice, policy, and future research

The results of this review offer several implications for practice, policy, and future research.

#### 4.10.1 Practice

The approaches discussed show potential to support clinicians in diagnosing, screening, and managing PDA, particularly in resource-limited settings with limited specialist expertise. Enhanced diagnostic accuracy and earlier identification of hemodynamically significant PDA may reduce delays in intervention and associated complications in preterm neonates. AI-based risk factor identification may inform the development of preventive strategies and targeted public health initiatives. Additionally, identifying subpopulations and factors influencing treatment outcomes could aid in evaluating treatment efficacy and pave the way for more individualized management strategies. However, as noted by McAdams et al. (32) and Sharma et al. (27), the application of AI in neonatology and pediatric cardiology remains in its infancy. The included studies demonstrate feasibility rather than high performing, validated models ready for clinical integration.

#### 4.10.2 Policy

Standardizing definitions for PDA study groups, particularly hemodynamically significant PDA, is essential for improving study comparability and enabling model generalization—a need emphasized in the broader PDA literature and reinforced by this review. National and international neonatology associations have a key role to play in leading efforts toward such standardization, facilitating the development and validation of severity scores to guide evidence-based criteria that can serve both clinical decision-making and research comparability. To support broader and more representative studies, secure, ethically responsible data sharing should be promoted across institutions, whilst safeguarding patient privacy. The endorsement of open-source collaboration—through the sharing of models, code, and anonymized datasets—would accelerate innovation and enhance transparency. Regulatory frameworks should guide the clinical validation and approval of artificial intelligence models to establish reliability, mitigate biases, address fairness, and ensure widespread applicability before clinical adoption.

#### 4.10.3 Future research

Future research should prioritize reaching consensus on standardized definitions for PDA subgroup classification—particularly regarding what constitutes hemodynamic significance. Establishing clear and uniform criteria is essential for enabling comparability across studies and providing a reliable ground truth from which AI systems can learn. Given the pilot nature of the included studies, many were designed primarily to demonstrate feasibility and were therefore simplified in ways that limit clinical utility. Common comorbidities such as congenital heart defects and pulmonary hypertension were excluded, and tasks were narrowly defined—for example, focusing on binary PDA detection rather than clinically nuanced assessments. With feasibility now established, future work should shift toward addressing more clinically meaningful tasks while striving for improved model performance and broader applicability. Multicenter studies should be implemented to improve generalizability across diverse populations and clinical settings. Larger datasets are needed to reduce the risk of bias and enable advanced DL modeling techniques. Incorporating temporal data, such as changes in cardiac motion or clinical trends, could enhance model accuracy and provide better insights into PDA evolution. Model explainability techniques should be implemented to facilitate clinical trust and usability. External validation on datasets from other institutions is necessary to assess generalizability. Advanced methodologies, including foundation models, vision transformers, self-supervised learning, and unsupervised approaches, have the potential to leverage high-dimensional or unlabeled data effectively and should be explored. Combining multimodal data—such as imaging, clinical variables, and biomarkers—may improve diagnostic precision and treatment predictions. Future studies should address real-world conditions, including class imbalance and population heterogeneity, and evaluate a variety of artificial intelligence-driven solutions against traditional baselines. Ethical concerns, including bias, fairness, and equitable access, require attention, and research should adopt standardized reporting frameworks like the Minimum Information for Clinical Artificial Intelligence Modeling (MI-CLAIM) (41) to ensure transparency and comparability.

## 5 Conclusion

Patent ductus arteriosus represents a complex and heterogeneous clinical condition, characterized by dynamic physiology, evolving disease severity, and frequent comorbidities in a highly vulnerable population. The absence of standardized subgroup definitions and consensus on optimal management strategies has contributed to variability in clinical practice and a fragmented research landscape. Within this context, artificial intelligence has been proposed as a potential tool to support more consistent, data-driven decision-making through the integration of high-dimensional clinical information.

Current AI-based approaches in PDA research demonstrate feasibility for supporting diagnostic processes, risk assessment, and prediction of treatment-related outcomes. However, most studies remain exploratory and lack the methodological maturity required for clinical validation or implementation. Key limitations include a high risk of bias, small sample sizes, limited external validation, and reliance on data that may not adequately reflect real-world clinical complexity.

Meaningful progress will depend on the use of comparable and clinically well-defined study populations and larger, more representative sample sizes, ideally achieved through prospective multicenter collaboration. Systematic risk-of-bias assessment and external validation should become standard practice. AI models should target clinically meaningful tasks using data representative of routine clinical practice, with explicit attention to fairness and potential sources of bias. Ongoing benchmarking of state-of-the-art AI methodologies against appropriate baseline methods, alongside the exploration of advanced techniques such as temporal modeling and multimodal data integration, may further enhance predictive performance and clinical relevance. Transparent reporting, sharing of code and model weights, and data-sharing strategies that protect patient privacy are essential for reproducibility and independent validation. Equally, explainability should be embedded in model design to enable clinicians to appropriately evaluate AI outputs and integrate them into clinical workflows, with AI intended to support, rather than replace, clinical judgment.

While the studies reviewed represent important early contributions, existing AI models are not yet positioned to meaningfully influence PDA care. Feasibility has been demonstrated, but further progress will require methodology attentive to bias, external validation and a sustained focus on clinical applicability. Addressing current limitations in future work will be pivotal in guiding AI applications for PDA from early feasibility toward clinically relevant and ethically sound translation. Only through such efforts can the potential of this technology be responsibly realized for this complex and highly vulnerable patient population.

## Data Availability

The original contributions presented in the study are included in the article/Supplementary Material, further inquiries can be directed to the corresponding author.

## References

[1] Pugnaloni F Doni D Lucente M Fiocchi S Capolupo I. Ductus arteriosus in fetal and perinatal life. J Cardiovasc Dev Dis. (2024) 11(4):113. 10.3390/jcdd11040113 38667731 PMC11050351

[2] Wren JT McNamara PJ Gillam-Krakauer M. Contemporary perspectives on the patent ductus arteriosus in preterm neonates: a hemodynamics-driven approach. Curr Treat Options Pediatr. (2024) 10(3):147–65. 10.1007/s40746-024-00296-3

[3] Roșca I Constantin AT Popescu DE Jura AMC Miu A Turenschi A. Are we able to prevent neonatal readmission? A retrospective analysis from a pediatrics department in ploiești, Romania. Medicina (B Aires). (2024) 60(5):705. 10.3390/medicina60050705 PMC11123246 38792888

[4] Smith A EL-Khuffash AF. Defining “haemodynamic significance” of the patent ductus arteriosus: do we have all the answers? Neonatology. (2020) 117(2):225–32. 10.1159/000506988 32450558

[5] Benitz WE Backes CH. At a crossroads for early medical treatment of persistent patent ductus arteriosus in preterm infants. J Perinatol. (2024) 44(10):1534–7. 10.1038/s41372-024-02022-1 38918573

[6] Semberova J Sirc J Miletin J Kucera J Berka I Sebkova S Spontaneous closure of patent ductus arteriosus in infants ≤1500 g. Pediatrics. (2017) 140(2):e20164258. 10.1542/peds.2016-4258 28701390

[7] Tolia VN Powers GC Kelleher AS Walker MW Herrman KK Ahmad KA Low rate of spontaneous closure in premature infants discharged with a patent ductus arteriosus: a multicenter prospective study. J Pediatr. (2022) 240:31–36.e2. 10.1016/j.jpeds.2021.07.035 34293369

[8] McNamara PJ Jain A El-Khuffash A Giesinger R Weisz D Freud L Guidelines and recommendations for targeted neonatal echocardiography and cardiac point-of-care ultrasound in the neonatal intensive care unit: an update from the American society of echocardiography. J Am Soc Echocardiogr. (2024) 37(2):171–215. 10.1016/j.echo.2023.11.016 38309835

[9] Babla K Duffy D Dumitru R Richards J Kulkarni A. Repeatability of PDA diameter measurements on echocardiography. Eur J Pediatr. (2022) 181(1):403–6. 10.1007/s00431-021-04178-w 34184120

[10] Singh Y Chan B Noori S Ramanathan R. Narrative review on echocardiographic evaluation of patent ductus arteriosus in preterm infants. J Cardiovasc Dev Dis. (2024) 11(7):199. 10.3390/jcdd11070199 39057619 PMC11277213

[11] Kindler A Seipolt B Heilmann A Range U Rüdiger M Hofmann SR. Development of a diagnostic clinical score for hemodynamically significant patent ductus arteriosus. Front Pediatr. (2017) 5:280. 10.3389/fped.2017.00280 29312911 PMC5743666

[12] Gokulakrishnan G Kulkarni M He S Leeflang MM Cabrera AG Fernandes CJ Brain natriuretic peptide and N-terminal brain natriuretic peptide for the diagnosis of haemodynamically significant patent ductus arteriosus in preterm neonates. Cochrane Database Syst Rev. (2022) 12(12):CD013129. 10.1002/14651858.CD013129.pub2 36478359 PMC9730301

[13] Smith A El-Khuffash A. Patent ductus arteriosus clinical trials: lessons learned and future directions. Child Basel Switz. (2021) 8(1):47. 10.3390/children8010047 PMC7830584 33467401

[14] Mitra S Florez ID Tamayo ME Mbuagbaw L Vanniyasingam T Veroniki AA Association of placebo, indomethacin, ibuprofen, and acetaminophen with closure of hemodynamically significant patent ductus arteriosus in preterm infants: a systematic review and meta-analysis. JAMA. (2018) 319(12):1221–38. 10.1001/jama.2018.1896 29584842 PMC5885871

[15] Mosalli R Alfaleh K. Prophylactic surgical ligation of patent ductus arteriosus for prevention of mortality and morbidity in extremely low birth weight infants. Cochrane Database Syst Rev. (2008) 2008(1):CD006181. 10.1002/14651858.CD006181.pub2 18254095 PMC8916218

[16] Reese J Shelton EL Slaughter JC McNamara PJ. Prophylactic indomethacin revisited. J Pediatr. (2017) 186:11–14.e1. 10.1016/j.jpeds.2017.03.036 28396028 PMC5520627

[17] Kluckow M Jeffery M Gill A Evans N. A randomised placebo-controlled trial of early treatment of the patent ductus arteriosus. Arch Dis Child Fetal Neonatal Ed. (2014) 99(2):F99–104. 10.1136/archdischild-2013-304695 24317704

[18] Hundscheid T Onland W Kooi EMW Vijlbrief DC De Vries WB Dijkman KP Expectant management or early ibuprofen for patent ductus arteriosus. N Engl J Med. (2023) 388(11):980–90. 10.1056/NEJMoa2207418 36477458

[19] EL-Khuffash A Weisz DE McNamara PJ. Reflections of the changes in patent ductus arteriosus management during the last 10 years. Arch Dis Child Fetal Neonatal Ed. (2016) 101(5):F474–8. 10.1136/archdischild-2014-306214 27118761

[20] Jansen EJS Hundscheid T Onland W Kooi EMW Andriessen P de Boode WP. Factors associated with benefit of treatment of patent ductus arteriosus in preterm infants: a systematic review and meta-analysis. Front Pediatr. (2021) 9:626262. 10.3389/fped.2021.626262 33634058 PMC7899974

[21] Hundscheid T Jansen EJS Onland W Kooi EMW Andriessen P De Boode WP. Conservative management of patent ductus arteriosus in preterm infants—a systematic review and meta-analyses assessing differences in outcome measures between randomized controlled trials and cohort studies. Front Pediatr. (2021) 9:626261. 10.3389/fped.2021.626261 33718300 PMC7946967

[22] Mitra S Scrivens A von Kursell AM Disher T. Early treatment versus expectant management of hemodynamically significant patent ductus arteriosus for preterm infants. Cochrane Database Syst Rev. (2020) 12(12):CD013278. 10.1002/14651858.CD013278.pub2 33301630 PMC8812277

[23] Zonnenberg I de Waal K. The definition of a haemodynamic significant duct in randomized controlled trials: a systematic literature review. Acta Paediatr. (2012) 101(3):247–51. 10.1111/j.1651-2227.2011.02468.x 21913976

[24] Al-Turkait A Szatkowski L Choonara I Ojha S. Management of patent ductus arteriosus in very preterm infants in England and Wales: a retrospective cohort study. BMJ Paediatr Open. (2022) 6(1):e001424. 10.1136/bmjpo-2022-001424 36053632 PMC8928285

[25] Ambalavanan N Aucott SW Salavitabar A Levy VY. Committee on Fetus and Newborn, Section on Cardiology and Cardiac Surgery. Patent ductus arteriosus in preterm infants. Pediatrics. (2025) 155(5):e2025071425. 10.1542/peds.2025-071425 40288780

[26] Sehgal A McNamara PJ. International perspective on management of a patent ductus arteriosus: lessons learned. Semin Fetal Neonatal Med. (2018) 23(4):278–84. 10.1016/j.siny.2018.03.002 29534972

[27] Sharma P Beam K Levy P Beam AL. PD(AI): the role of artificial intelligence in the management of patent ductus arteriosus. J Perinatol. (2023) 43(2):257–8. 10.1038/s41372-023-01606-7 36646822 PMC12129057

[28] Ghorbani A Ouyang D Abid A He B Chen JH Harrington RA Deep learning interpretation of echocardiograms. Npj Digit Med. (2020) 3(1):10. 10.1038/s41746-019-0216-8 31993508 PMC6981156

[29] Maturi B Dulal S Sayana SB Ibrahim A Ramakrishna M Chinta V Revolutionizing cardiology: the role of artificial intelligence in echocardiography. J Clin Med. (2025) 14(2):625. 10.3390/jcm14020625 39860630 PMC11766369

[30] Kelly CJ Karthikesalingam A Suleyman M Corrado G King D. Key challenges for delivering clinical impact with artificial intelligence. BMC Med. (2019) 17(1):195. 10.1186/s12916-019-1426-2 31665002 PMC6821018

[31] Markus AF Kors JA Rijnbeek PR. The role of explainability in creating trustworthy artificial intelligence for health care: a comprehensive survey of the terminology, design choices, and evaluation strategies. J Biomed Inform. (2021) 113:103655. 10.1016/j.jbi.2020.103655 33309898

[32] McAdams RM Kaur R Sun Y Bindra H Cho SJ Singh H. Predicting clinical outcomes using artificial intelligence and machine learning in neonatal intensive care units: a systematic review. J Perinatol. (2022) 42(12):1561–75. 10.1038/s41372-022-01392-8 35562414

[33] Lei H Ashrafi A Chang P Chang A Lai W. Patent ductus arteriosus (PDA) detection in echocardiograms using deep learning. Intell Based Med. (2022) 6:100054. 10.1016/j.ibmed.2022.100054

[34] Na JY Kim D Kwon AM Jeon JY Kim H Kim CR Artificial intelligence model comparison for risk factor analysis of patent ductus arteriosus in nationwide very low birth weight infants cohort. Sci Rep. (2021) 11(1):22353. 10.1038/s41598-021-01640-5 34785709 PMC8595677

[35] El-Khuffash A James AT Corcoran JD Dicker P Franklin O Elsayed YN A patent ductus arteriosus severity score predicts chronic lung disease or death before discharge. J Pediatr. (2015) 167(6):1354–1361.e2. 10.1016/j.jpeds.2015.09.028 26474706

[36] Umapathi KK Muller B Sosnowski C Thavamani A Murphy J Awad S A novel patent ductus arteriosus severity score to predict clinical outcomes in premature neonates. J Cardiovasc Dev Dis. (2022) 9(4):114. 10.3390/jcdd9040114 35448090 PMC9033137

[37] Page MJ McKenzie JE Bossuyt PM Boutron I Hoffmann TC Mulrow CD The PRISMA 2020 statement: an updated guideline for reporting systematic reviews. Br Med J. (2021) 372:n71. 10.1136/bmj.n71 33782057 PMC8005924

[38] Sridharan K Doss C GP Cathryn R H Kumar D T Al Jufairi M. Comparative analysis of machine learning algorithms evaluating the single nucleotide polymorphisms of metabolizing enzymes with clinical outcomes following intravenous paracetamol in preterm neonates with patent ductus arteriosus. Curr Drug Metab. (2024) 25(2):128–39. 10.2174/0113892002289238240222072027 38445694

[39] Wolff RF Moons KGM Riley RD Whiting PF Westwood M Collins GS PROBAST: a tool to assess the risk of bias and applicability of prediction model studies. Ann Intern Med. (2019) 170(1):51–8. 10.7326/M18-1376 30596875

[40] JBI. Chapter 7: Systematic reviews of etiology and risk. In: *JBI Manual for Evidence Synthesis*. JBI. (2020). Available online at: https://jbi-global-wiki.refined.site/space/MANUAL/355863557/Previous+versions?attachment=/download/attachments/355863557/JBI_Reviewers_Manual_2020June.pdf&type=application/pdf&filename=JBI_ Reviewers_Manual_2020June.pdf#page=217 (Accessed 17 June 2025).

[41] Norgeot B Quer G Beaulieu-Jones BK Torkamani A Dias R Gianfrancesco M Minimum information about clinical artificial intelligence modeling: the MI-CLAIM checklist. Nat Med. (2020) 26(9):1320–4. 10.1038/s41591-020-1041-y 32908275 PMC7538196

[42] Gómez-Quintana S Schwarz CE Shelevytsky I Shelevytska V Semenova O Factor A A framework for AI-assisted detection of patent ductus arteriosus from neonatal phonocardiogram. Healthcare. (2021) 9(2):169. 10.3390/healthcare9020169 33562544 PMC7914824

[43] Park S Moon J Eun H Hong JH Lee K. Artificial intelligence-based diagnostic support system for patent ductus arteriosus in premature infants. J Clin Med. (2024) 13(7):2089. 10.3390/jcm13072089 38610854 PMC11012712

[44] Erno J Gomes T Baltimore C Lineberger JP Smith DH Baker GH. Automated identification of patent ductus arteriosus using a computer vision model. J Ultrasound Med. (2023) 42(12):2707–13. 10.1002/jum.16305 37449663

[45] Chang P Choi HS Lee J Kim HH. Extraction and evaluation of features of preterm patent ductus arteriosus in chest x-ray images using deep learning. Sci Rep. (2024) 14(1):29382. 10.1038/s41598-024-79361-8 39592675 PMC11599863

[46] Liu TX Zheng JX Chen Z Zhang ZC Li D Shi LP. An interpretable machine-learning model for predicting the efficacy of nonsteroidal anti-inflammatory drugs for closing hemodynamically significant patent ductus arteriosus in preterm infants. Front Pediatr. (2023) 11:1097950. 10.3389/fped.2023.1097950 37082702 PMC10110971

[47] Sharma P Gearhart A Luo G Palepu A Wang C Mayourian J Development and validation of a novel deep learning model to predict pharmacologic closure of patent ductus arteriosus in premature infants. J Am Soc Echocardiogr. (2025) 38(7):S0894731725002093. 10.1016/j.echo.2025.03.018 PMC12229758 40220935

[48] Jura AMC Popescu DE Cîtu C Biriș M Pienar C Paul C Predicting risk for patent ductus arteriosus in the neonate: a machine learning analysis. Medicina (B Aires). (2025) 61(4):603. 10.3390/medicina61040603 PMC12028894 40282894

[49] Matsushita FY Krebs VLJ De Carvalho WB. Identifying two distinct subphenotypes of patent ductus arteriosus in preterm infants using machine learning. Eur J Pediatr. (2023) 182(5):2173–9. 10.1007/s00431-023-04882-9 36853570

[50] Zhang SY Zhang YD Li H Wang QY Ye QF Wang XM Explainable machine learning model for predicting decline in platelet count after interventional closure in children with patent ductus arteriosus. Front Pediatr. (2025) 13:1519002. 10.3389/fped.2025.1519002 39981204 PMC11839778

[51] Reddy CD Lopez L Ouyang D Zou JY He B. Video-Based deep learning for automated assessment of left ventricular ejection fraction in pediatric patients. J Am Soc Echocardiogr. (2023) 36(5):482–9. 10.1016/j.echo.2023.01.015 36754100

[52] Sehgal A Paul E Menahem S. Functional echocardiography in staging for ductal disease severity : role in predicting outcomes. Eur J Pediatr. (2013) 172(2):179–84. 10.1007/s00431-012-1851-0 23052621

[53] Fink D El-Khuffash A McNamara PJ Nitzan I Hammerman C. Tale of two patent ductus arteriosus severity scores: similarities and differences. Am J Perinatol. (2018) 35(1):55–8. 10.1055/s-0037-1605576 28787748

[54] Giesinger RE Hobson AA Bischoff AR Klein JM McNamara PJ. Impact of early screening echocardiography and targeted PDA treatment on neonatal outcomes in “22–23” week and “24–26” infants. Semin Perinatol. (2023) 47(2):151721. 10.1016/j.semperi.2023.151721 36882362

[55] Masutani S Isayama T Kobayashi T Pak K Tomotaki S Iwami H Generation of PLASE score for patent ductus arteriosus using the PLASE study database. Pediatr Res. (2025) 98:152–60. 10.1038/s41390-025-03803-w 39922923 PMC12411220

[56] Gearhart A Elrod M Gomes T Golbus A Baltimore C Wakser C Abstract 4135928: externally validated deep learning model for patent ductus arteriosus detection by echocardiography in preterm infants. Circulation. (2024) 150(Suppl_1):A4135928. 10.1161/circ.150.suppl_1.4135928

